# A Format for Phylogenetic Placements

**DOI:** 10.1371/journal.pone.0031009

**Published:** 2012-02-22

**Authors:** Frederick A. Matsen, Noah G. Hoffman, Aaron Gallagher, Alexandros Stamatakis

**Affiliations:** 1 Program in Computational Biology, Fred Hutchinson Cancer Research Center, Seattle, Washington, United States of America; 2 Laboratory Medicine, University of Washington, Seattle, Washington, United States of America; 3 Heidelberg Institute for Theoretical Studies, Heidelberg, Germany; J. Craig Venter Institute, United States of America

## Abstract

We have developed a unified format for *phylogenetic placements*, that is, mappings of environmental sequence data (e.g., short reads) into a phylogenetic tree. We are motivated to do so by the growing number of tools for computing and post-processing phylogenetic placements, and the lack of an established standard for storing them. The format is lightweight, versatile, extensible, and is based on the JSON format, which can be parsed by most modern programming languages. Our format is already implemented in several tools for computing and post-processing parsimony- and likelihood-based phylogenetic placements and has worked well in practice. We believe that establishing a standard format for analyzing read placements at this early stage will lead to a more efficient development of powerful and portable post-analysis tools for the growing applications of phylogenetic placement.

## Introduction

“Phylogenetic placement” has become popular in the last several years as a way to gain an evolutionary understanding of a large collection of sequences. The input to a phylogenetic placement algorithm consists of a reference tree, a corresponding reference multiple sequence alignment, and a collection of query sequences. The output of a phylogenetic placement algorithm is a set of assignments of the query sequences to branches of the tree; there is at least one such assignment for each query. A query can be assigned to more than one branch on the reference tree to express placement uncertainty for that query sequence.

Phylogenetic placement methods circumvent several problems associated with applying traditional phylogenetic algorithms to large, environmentally-derived sequence data. The computational burden is decreased compared to constructing a tree containing reference and query sequences *de novo*, resulting in algorithms that can place thousands to tens of thousands of query sequences per hour *and* per processing core into a reference phylogeny with one thousand taxa. Because computation is performed on each query sequence individually and independently, placement algorithms are also straightforward to parallelize. The relationships between the query sequences are not investigated. Hence, the size of the search space is reduced from an exponential to just a linear number of phylogenetic hypotheses. Moreover, short and/or non-overlapping query sequences pose less of a problem, as query sequences are compared to the full-length reference sequences. Visualization of samples and comparison between samples are facilitated by the assumption of a fixed reference tree, that can be drawn in a way which highlights the location and distribution of reads.

The advent of high-throughput sequencing has motivated a growing interest in phylogenetic placement. The basic idea is as old as computational phylogenetics [1], [2] although these insertions historically have been considered as just the first step towards full *de novo* tree reconstruction. Recent implementations have focused on algorithms for likelihood-based placement, such as [3], [4], with more efficient recent implementations [5]–[7]. These tools are being incorporated into popular workflows for microbial ecology, such as QIIME [8] and the next version of AMPHORA [9]. Comparative methods are being developed and implemented in software [10], [11], and work is underway to extend a tree viewer [12] to visualize placements. Dedicated algorithms to align reads with respect to reference alignments for subsequent phylogenetic placement are also being developed [13], [14].

Because of this expansion of activity, standards are needed. The original versions of pplacer [6] and EPA [5] each implemented their own idiosyncratic tabular file formats. These ad-hoc formats kept post-analysis tools from being interoperable and hindered tool comparison.

In this letter, we describe a lightweight file format that will ensure consistency between tools. Because it adopts JSON (JavaScript Object Notation) [15], a widely used data interchange standard, and extends the widely used Newick format for phylogenetic trees, it is straight-forward to parse using existing tools. It can be used with likelihood, posterior probability, and parsimony-based placements, can associate an arbitrary number of sequence names associated with a placement, and can store a generalization of a name list called a *named multiplicity* as described below. Basic operations such as subsetting arbitrary collections of placements and merging these lists are easily done. The format can be extended to incorporate additional information, such as taxonomic assignments.

Although we have made our best efforts to ensure that the format is sufficiently extensible without changing the specification, it may be necessary to change it in the future. For that reason, the authoritative version of the file format will be maintained online at http://arxiv.org/abs/1201.3397. The version described in this document is version 3 of the file format.

## Results and Discussion

### Concepts

We first establish terminology in order to describe the placement format. As described above, phylogenetic placement is performed by inserting a collection of query sequences onto a fixed reference tree in order to optimize a given criterion. Specifically, for a given set of query sequences the objective is to find an attachment of each query sequence to the tree that maximizes likelihood or minimizes the parsimony score for the reference tree with that (and only that) query sequence attached. Because each query sequence is placed individually on the tree, the run time complexity is of order the product of the number of reference sequences, the number of query sequences, and the number of sites in the alignment.

There may be more than one good or likely location for a query sequence, and it is important to record this uncertainty. Uncertainty may be expressed in terms of placement locations that have equal parsimony scores, in terms of *likelihood weight ratio* (the ratio of likelihoods of the various placements), or in terms of posterior probability. Because a given query sequence thus can be considered to have a collection of placements with varying certainties, we use the word *pquery* for “placed query” to denote the collection of placements of a single query sequence.

It is also common to obtain several identical sequence reads from deep-sequencing studies. Furthermore, closely related sequences may exhibit such similar placement results that a user may wish to group them together for ease of analysis. For this reason, we allow more than one sequence name to be associated with a given pquery.

Users may simply wish to keep the number of sequences associated with a given pquery instead of the complete collection of names. More generally, they may wish to simply have a single floating point number, the *multiplicity* associated with a pquery. This multiplicity may represent a transformed measure of the quantity of sequences associated with that pquery, analogous to the transforms that are commonly applied to ecological count data. For that reason, we also allow the specification of a named multiplicity associated with a pquery in place of a list of names.

### Design

One possible representation of a collection of placements would be a single tree with each placement inserted as a pendant branch. That design is problematic for representations of uncertainty; if each possible location for every query sequence were represented as a pendant branch, then it would be difficult to distinguish the pendant edges that resulted from uncertainty with those resulting from multiple query sequences. Subsetting collections of placements would require tree “surgery”. Furthermore, packing everything into a tree would make placement-specific metadata such as multiple confidence measures difficult to keep track of. Also, visualizing a reference tree with 1,000 taxa and 10,000 queries *and* with several placements per query may become computationally and visually cumbersome.

These considerations led us to develop a format where the placements are represented as a list, and their branch assignments are indexed by numbered edges of the reference tree. Each placement is associated with entries for a collection of *fields*, which can contain arbitrary data about the placement. With such a list-based format, subsetting pqueries becomes trivial.

With the separation of reference set and placements in mind, our goals in designing the format were: to adopt a popular extensible open standard human-readable file format, to ease parsing between languages and tools, and to deploy a light-weight format that can handle large collections of placements on large reference trees without requiring too much space. We chose JSON, since it satisfies all of the above criteria.

Using the JSON syntax, one option would be to individually associate each placement with an arbitrary collection of information using key-value pairs for each placement. However, doing so would have created a substantial file size overhead, as the total number of characters used to represent the keys would be about the same as the total number of characters used to represent the data. Because of this, the field titles are written out only once, and every placement just supplies the data as an array with entries in the correct corresponding order, as described below.

## Methods

### Specification

Files using the format described in this paper will use the .jplace suffix, which is short for JSON placement.

The basic types in a JSON file are Array, Boolean, Number, Object, String, and null. These are familiar terms except Object, which is a list of colon separated key-value pairs, where the keys are strings and the values are arbitrary types. A JSON file contains a single JSON object.

In .jplace files, the fundamental object contains a list of four keys: tree, fields, placements, metadata, and version. We will describe each of these in succession, but this need not correspond to their order in the JSON object. Indeed, the order of key-value pairs in a JSON object is unspecified.

#### Tree

To represent the tree, we extend the well-known Newick file format. In that format, commas and parentheses are used to display the structure of the tree. The taxon names (leaf labels) are inserted as plain text. It is also common to label internal nodes with strings appearing after the closing of a parenthesis. It is also possible to label edges of the tree with strings enclosed in square brackets. For example, the tree

((A:.01[e], B:.01)D:.01[g], C:.01[h]);

is a tree with some edge labels and some node labels.

We extend this format with edge numberings in curly braces:

((A:.01[e]{0}, B:.01{1})D:.01{3}[g], C:.01{4}[h]) {5};

These edge numberings index the edges for the placements. We use curly braces to distinguish between our edge numberings and other edge label values such as posterior probability or bootstrap branch (bipartition) support.

Although not required for parsing, we use a convention that placement algorithms should use a pre-defined edge numbering. Specifically, we enforce that branches are labeled by a depth-first traversal (descending left subtree first and starting at the root/top-level node in the reference input tree) and we assign branch numbers by a post-order traversal. This strict definition is convenient to ensure one-to-one comparability of results obtained from various placement algorithms.

We also require the output tree to be identical as a planar tree to the input reference tree, that is, the subtree ordering and top-level multifurcation must remain unchanged. In the case of parsimony-based placements, the reference tree may optionally be represented without branch lengths.

#### Fields

The value associated with fields is an array of strings specifying the headers in the same order as the arrays of placement data. For example, the default fields for a maximum likelihood EPA or pplacer run are edge_num, likelihood, like_weight_ratio, distal_length, and pendant_length.

The edge_num specifies the placement edge, and is necessary for all placements. The pendant_length is the branch length for the placement edge, and distal_length is the length from the distal (away from the root) side of the reference tree edge to the placement attachment location. The likelihood is the likelihood of the tree with the placement attached, which may be calculated from an alignment with columns masked out that do not appear in the read. For that reason, the log likelihood of the placement may be better (closer to zero) than the log likelihood of the reference tree on the full-length alignment. The like_weight_ratio is the ratio of that placement's likelihood to that of the other alternate placements for that read. For a pplacer posterior probability run, the marginal likelihood marginal_prob and the posterior probability post_prob are also included.

In contrast to pplacer, EPA optimizes three branch lengths associated with a placement: the pendant branch length, the distal branch length, and the proximal branch length. Thus, the EPA output could be extended to comprise the full information generated by the EPA algorithm by adding a proximal_length field. Because the currently available downstream placement analysis tools (e.g., guppy) do not use this additional information, it is not included in the EPA .jplace output file at present.

The corresponding fields for parsimony-based placements (currently only available in EPA) are edge_num and parsimony. The parsimony field just contains the parsimony score of the placement as an integer.

#### Placements

The value associated with the placements key is the list of placements grouped into pqueries. The representation of each pquery is a JSON object of its own, with two keys: p, for placements, and either n for names or nm for names with multiplicity. The value associated with p is the list of placements for that pquery with entries corresponding to the fields in the order set up by the fields described above. The list of placements shows possible placement locations along with their confidence scores and other information. The value associated with n is a list of names associated with that pquery. Although an arbitrary list of names can be associated with a pquery, the typical use will be to collect placement information for identical or closely related sequences. The value associated with nm is a list of *named multiplicities*, which are simply ordered pairs of a name and then a positive floating point value. As described above, multiplicity can be used to keep track of the number of sequences associated with that name or a transform thereof.

For parsimony-based placements we require all equally parsimonious placements of a query to be included in the output file. This is to enable easy comparison between parsimony-based placement methods; if only one of the best-scoring placements is arbitrarily selected in one way or another, comparing programs based on our standard will become error-prone and biased.

### Other keys

There are also two other keys in the fundamental JSON object. The first, version, is mandatory, and indicates an integer version number of the format. The version described in this paper is 3. The second, metadata, is optional and keys an arbitrary object for metadata. It can describe how the placement file was generated, which phylogenetic model was used, and so on. In EPA and pplacer we include the full command line string of the placement program invocation to allow for easy reproducibility of results.

### A small example

{

 “tree”: “((A:0.2{0},B:0.09{1}):0.7{2},C:0.5{3}){4};”,

 “placements”:

 [

  {“p”:

   [[1, −2578.16, 0.777385, 0.004132, 0.0006],

   [0, −2580.15, 0.107065, 0.000009, 0.0153]

   ],

  “n”: [“fragment1”, “fragment2”]

  },

  {“p”: [[2, −2576.46, 1.0, 0.003555, 0.000006]],

   “nm”: [[“fragment3”, 1.5], [“fragment4”, 2]]}

 ],

 “metadata”:

 {“invocation”:

  “pplacer -c tiny.refpkg frags.fasta”

 },

 “version”: 3,

 “fields”:

 [“edge_num”, “likelihood”, “like_weight_ratio”,

    “distal_length”, “pendant_length”]

}

### Tabular representation

The JSON object can be readily transformed into a tabular format to more easily summarize or explore the data using statistical tools or a relational database. With the addition of an index (placement_id) to form a relation between placements and sequence names, two tables are sufficient: one with columns placement_id followed by each of the fields contained by each pquery array, and another providing a mapping of every placement_id to each of the corresponding sequence names or named multiplicities. This transformation can be performed efficiently using any modern high level language with a JSON parsing library. Such a representation of the data is useful for supporting analyses that involve grouping and partitioning placements and sequences.

The latest versions of EPA (http://github.com/stamatak/standard-RAxML) and pplacer (http://matsen.fhcrc.org/pplacer/) both produce these files. The guppy program in the pplacer suite has a number of subcommands that allow transformations and filterings of these files (manuscript in preparation). MePal, an implementation of placement using an alignment-free generalization to indels of Felsenstein's phylogenetic pruning algorithm [16], now imports and writes out this format as well. The TopiaryExplorer [12] tree visualization package is now in the process of being extended to read this format for visualization.
